# The Role of Artificial Intelligence in Advancing Theranostics Dosimetry for Cancer Therapy: a Review

**DOI:** 10.1007/s13139-025-00939-9

**Published:** 2025-08-23

**Authors:** Sang-Keun Woo

**Affiliations:** 1 Division of applied RI, Korea Institute of Radiological and Medical Sciences, 75, Nowon-ro, Nowon-gu, Seoul, Republic of Korea; 2 Radiological & Medical Sciences, University of Science & Technology, Daejeon, Korea

**Keywords:** Deep learning, CNN, Generative adversarial network, Personalized dosimetry, Image-based dosimetry, Voxel-based dose prediction, Medical image synthesis

## Abstract

Cancer treatment has greatly benefited from advancements in radiopharmaceutical therapy, which requires precise dosimetry to enhance therapeutic efficacy and minimize risks to healthy tissues. This review investigated the role of artificial intelligence (AI) in theranostic radiopharmaceutical dosimetry, focusing on image quality enhancement, dose estimation, and organ segmentation. An in-depth review of the literature was conducted using targeted keywords searches in Google Scholar, PubMed, and Scopus. Selected studies were evaluated for their methodologies and outcomes. Traditional dosimetry techniques such as organ-level and voxel-based methods are discussed. Deep learning (DL) models based on U-Net, generative adversarial networks, and hybrid transformer networks for image synthesis and generation, image quality improvement, organ segmentation, and radiation dose estimation are reviewed and discussed. While DL shows great potential for enhancing dosimetry accuracy and efficiency, challenges such as the need for accurate dose estimation from theranostic pairs, lack of imaging data, and modeling of radionuclide decay chains must be addressed using DL models. In addition, the optimization and standardization of DL and AI models is crucial for ensuring clinical reliability and should be given high priority to support their effective integration into clinical practice.

##  Introduction

Cancer is characterized by the uncontrolled growth and spread of abnormal cells and remains a major global health challenge. The estimated number of new cancer cases worldwide is projected to increase by 75% rising from 20 million in 2022 to approximately 35 million by 2050 [1]. Various types of cancers are treated with radiation therapy, which employs ionizing radiation to destroy cancerous cells while sparing healthy tissues. Radiation therapy encompasses external beam therapy or radiopharmaceutical therapy (RPT). External beam radiation therapy is one of the most popular cancer treatments and is commonly performed using linear accelerator, which directs high-energy X-rays, protons, or electron beams to the cancer site.

Advances in medicine have also highlighted the importance of radiopharmaceuticals in cancer diagnosis and treatment. Radiopharmaceuticals are specialized compounds labeled with radioactive isotopes that target specific cellular structures and deliver cytotoxic radiation to tumor cells, enabling the precise targeting of cancer cells. Cancer treatment using radiopharmaceutical is often called RPT or targeted radionuclide therapy (TRT). Another advantage of radiopharmaceuticals is that they can be imaged directly or via surrogate imaging using techniques such as positron emission tomography (PET) or single-photon emission computed tomography (SPECT) [2]– [3]. These images can be used to evaluate therapeutic outcomes, enable image-based dosimetry, and diagnose diseases. The therapeutic use of radiopharmaceuticals began in the 1940 s [4], when radioactive iodine was first employed for the treatment of thyroid disease. Many radiopharmaceuticals labeled with different radioisotopes have since been developed to target various types of cancers with high therapeutic efficacy [5–13].

Several therapeutic radiopharmaceuticals have been approved by the food and drug association such as ^131^I-MIBG (AZEDRA^®^) for the treatment of pheochromocytoma or paraganglioma [14], [^177^Lu]Lu-DOTA-TATE LUTATHERA^®^) for somatostatin receptor-positive gastroenteropancreatic neuroendocrine tumors [15], [^177^Lu] Lu-PSMA-617 (Pluvicto™) for prostate-specific membrane antigen (PSMA)-positive metastatic castration-resistant prostate cancer [16], and [^223^Ra]RaCl_2_ (Xofigo^®^) for castration-resistant prostate cancer and symptomatic bone metastases [17]. Delivering the highest possible absorbed dose to the tumor while minimizing the dose to healthy tissues is essential for achieving the highest therapeutic efficacy. Despite the great therapeutic efficacy of radiopharmaceuticals, estimating the absorbed dose is essential for establishing dose-response relationships in clinical RPT, thus enabling the evaluation of therapeutic outcomes and associated toxicities [18]. Therefore, improved dosimetry techniques are required to accurately assess the risks and benefits of radiopharmaceuticals in clinical applications. This paper provides an in-depth review of conventional dosimetry approaches and the evolving role of AI in enhancing RPT dosimetry across various tasks.

##  Dosimetry Techniques and Tools

Internal dosimetry is commonly performed using the methodology introduced by the Medical Internal Radiation Dose (MIRD) committee [19]. Initially, this approach was based on organ-level dose estimation using organ time integral activity and organ S-value, which are the mean absorbed dose to the target organ per unit activity in the source region. OLINDA/EXM was the first and most widely used dosimetry software based on the MIRD approach. However, organ-level dosimetry calculates the mean absorbed dose assuming a uniform activity distribution of radiopharmaceuticals in the target organ or tissue. Additionally, organ-level dosimetry does not consider patient-specific anatomy. Instead, it relies on standardized anatomical models such as reference phantoms to estimate the radiation dose. These models represent the average anatomical features and organ geometries, which may differ from the anatomy of individual patients [20]. Several software packages, such as AIDE [21], DCAL [22], IDAC-DOSE [23], MIRDcalc [24], and MIRDOSE [25] have been developed to calculate doses at the organ level.

To address these challenges, voxel-based dosimetry has been introduced as a more precise method. Voxel-based dosimetry is reportedly superior to the mean-absorbed dose approach for establishing an absorbed dose-effect relationship in TRT [26]. This approach allows the calculation of the absorbed dose on a voxel-by-voxel basis, providing three-dimensional dose maps. Utilizing quantitative imaging data from modalities such as SPECT or PET combined with anatomical information from computed tomography (CT) or magnetic resonance imaging (MRI), voxel-based dosimetry accounts for heterogeneities in radiopharmaceutical uptake and patient-specific anatomical variations. This technique also includes several methods, such as the direct Monte Carlo (MC) method, dose kernel convolution (DKC), and direct voxel S-value(VSV).

The direct MC method incorporates heterogeneities in both the activity distribution and tissue properties. This technique simulates particle transport using MC engines and calculates the energy deposition at the voxel level. The direct MC method is considered to be the gold standard for accurate dose estimation. Several MC-based software packages such as VIDA [27], RAYDOSE [28], SIMDOSE [29], 3D-RAD [30], and OEDIPE [31] have been developed. However, the direct MC method is not used in routine clinical practice because of its time consumption and computational demands.

Other methods such as the dose point kernel (DPK) [32] and VSV [33] approaches have been proposed to speed up computation and address the nonuniform distribution of radiopharmaceuticals in organs. The DPK method represents the radially absorbed dose distribution around an isotropic point source in a homogeneous aqueous medium. Graves et al. provided dose-point kernels for 2,174 radionuclides [34]. Alternatively, VSV extends the organ-based MIRD schema to the voxel level by defining sources and targets as voxels with voxel-specific S-value precomputed for various isotopes and voxel sizes. Unlike DPK, VSV does not require computationally intensive conversions of spherical to Cartesian coordinates, but relies on tabulated S-value for each radionuclide. However, both methods assume uniform tissue density using fast Fourier or Hartley transforms [35, 36]. Table 1 summarizes the advantages and disadvantages of each dosimetry method. QDOSE (ABX-CRO advanced pharmaceutical services Forschungsgesellschaft mbH) is an advanced molecular imaging dosimetry software designed for internal radiation dose assessments at both the voxel (voxel S kernels) and organ (integrated with IDAC-Dose 2.1) level. The software supports AI-based semi- and fully-automated organ segmentation, single time-point dosimetry, and one-click hybrid dosimetry, offering precision and efficiency in dosimetric analysis. Another software package for voxel-level dosimetry is MIM (MIM Software, Inc., Cleveland, OH, USA), which supports single time-point and voxel-level dosimetry. VoxelDose [37], BigDose [38], and RMDP [39] are additional software packages based on voxel-based dosimetry.

**Table 1:** Comparison between organ-based dosimetry, voxel-based dosimetry and direct MC method

	Organ-based dosimetry	Voxel-based dosimetry	Direct MC method
Resolution	Provides average dose per organ	Provides the spatial distribution of the dose within the organ	Provides the spatial distribution of the dose within the organ
Computation Complexity	Relatively simple, uses mean kinetic parameters	Less than direct MC method	Computationally intensive. Requires voxel-by-voxel modeling
Accuracy	Limited by assumption of the uniform activity distribution	Limited by assumption of the homogenous medium density	High accuracy (gold standard)
Activity distribution	Homogeneous activity distributions	Heterogeneous activity distribution	Heterogeneous activity distribution
Medium density	Homogenous density	Homogenous density (water)	Heterogeneous density
Time Requirements	Rapid	Less time-consuming than the direct MC method	Slow

*MC* Monte Carlo

## AI Roles for Enhancing Dosimetry in RPT: Image synthesis, generation, and Quality Improvement

In radiopharmaceutical dosimetry, AI, particularly DL, has emerged as a transformative tool for improving the accuracy and efficiency of dosimetric assessments. Beyond direct dose estimation and the generation of high-resolution dose maps, DL models have been effectively employed in critical preprocessing steps, such as medical image enhancement, image generation, and organ segmentation, which play a crucial role in improving the precision of dose calculations. Using DL techniques to synthesize particular medical images using different modalities (e.g., PET-CT or MRI-CT) [40–47] or the same modality, has the potential to significantly reduce the risk of additional radiation exposure. Furthermore, DL has shown excellent ability to enhance image quality via noise reduction and super-resolution modeling to generate clinically clear images with improved quality and detail. The following sections describe the use of several DL architectures [48–52] for PET image generation/synthesis and quality enhancement.

###  PET Image Synthesis/generation Using AI

**Fig. 1:**
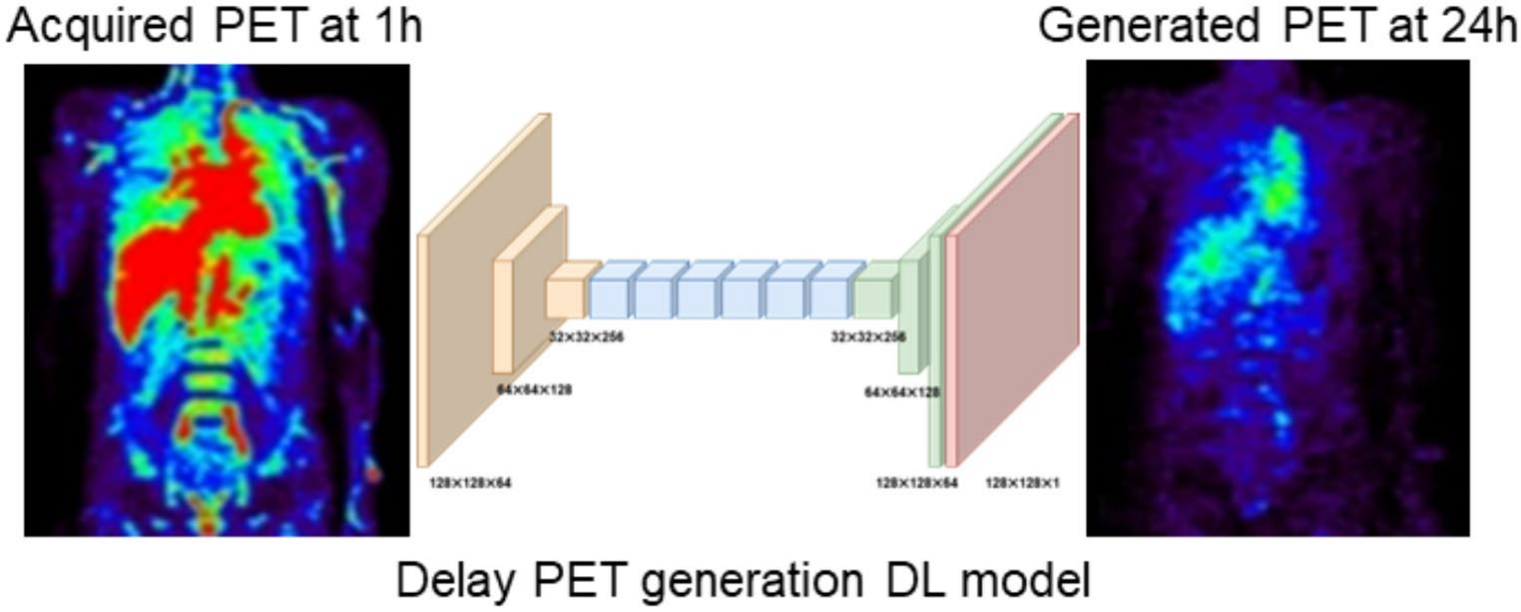
Conceptional graphic of delayed time PET image synthesis

In RPT, PET images are commonly used for image-based dosimetry assessment because of the high resolution and sensitivity of PET imaging. However, one of the primary limitations is the use of short-lived radionuclides, which limits the imaging time and demands rapid coordination between radiotracer production and patient administration [53]. Therefore, the use of AI for generating new or delayed PET images (Fig. 1) from early acquired images has the potential to overcome this limitation and reduce radiation exposure. Jyoti et al. proposed a generative adversarial network (GAN)-based model for synthesizing brain PET images representing three stages of Alzheimer’s disease: normal control (NC), mild cognitive impairment, and Alzheimer’s disease (AD) [54]. The model was trained separately for each stage using real PET images and noise samples. The synthetic images were evaluated using the peak signal-to-noise ratio (PSNR) and structural similarity index (SSIM), achieving a PSNR of 32.83 and SSIM of 77.48, indicating strong visual similarity to real PET scans. Qualitative assessments and a classification task using a 2D Convolutional Neural Networks (2D-CNN) showed that models trained with the synthetic data achieved improved diagnostic performance. Wang et al. investigated the feasibility of using deep learning to generate synthetic PET images of synaptic density (¹¹C-UCB-J) and amyloid deposition (¹¹C-PiB) from more widely available ¹⁸F-FDG PET scans [55]. Utilizing a 3D U-Net architecture, four models were trained to predict the standardized uptake value ratio (SUVR) and distribution volume ratio (DVR) of ¹¹C-UCB-J using different inputs derived from ¹⁸F-FDG scans (SUVR and Ki ratio). The models were trained and tested on data from 54 participants (21 CN, 33 with AD). Evaluation using normalized root mean square error (NRMSE), SSIM, and Pearson’s correlation showed satisfactory results, with mean region-of-interest biases mostly within ± 2% across the AD and CN groups. Although the ¹¹C-PiB SUVR prediction was more challenging, the study demonstrated that incorporating additional diagnostic or clinical information could help to reduce bias to < 5% in most regions. Overall, this work supports the potential of deep learning to synthesize high-value PET modalities from routine tracers.

While most studies focus on generating new synthetic PET images for new patients or on transforming images from a different modality, Kim et al. developed a DL-based method to synthesize delayed [¹⁸F]FDG PET images from early scans, aiming to reducing the need for multiple time-point acquisitions [56]. Eighteen healthy participants underwent PET imaging at 5, 14, 31, and 52 min post-injection. A paired image-to-image (I2I) translation framework based on GAN with a U-Net-based generator and a PatchGAN discriminator was used. The mode showed high capability for preserving image details, achieving a PSNR of 53.29 dB and an Fréchet inception distance (FID) score of 21.36 when predicting 52-min images from 14-min scans. The translation accuracy improved with the time gap between early and delayed scans. Furthermore, the organ-level mean standardized uptake values of generated images showed good agreement with the ground truth for the muscle, heart, liver, and spleen (errors < 0.2), although the model underperformed for the kidneys and bladder due to individual variability and dynamic uptake. Regardless, this study demonstrates the feasibility of using GAN-based I2I translation to synthesize delayed time PET images. Another study by Kim et al. synthesized delayed PET images of [⁶⁴Cu]DOTA-rituximab from early-time scans [57]. PET scans from six patients with lymphoma were acquired at 1, 24, and 48 h post-injection. A paired I2I translation framework based on the Pix2Pix GAN architecture, using a U-Net-like generator with residual blocks and a PatchGAN discriminator was used. The model showed SSIM and FID values of 0.8094 and 62.93 for generating PET images at 24 h post-injection and 0.7714 and 74.35 for synthesized PET images at 48 h post-injection, respectively. Furthermore, organ-level dosimetry using new synthesized images was in good agreement with real acquired images. The study demonstrates that GAN-based image synthesis can potentially reduce the need for multiple imaging points in radiopharmaceutical radioimmunoconjugates (RIC) dosimetry, streamlining the clinical workflow and improving patient convenience.

###  PET Image Denoising/reconstruction Using AI

**Fig. 2:**
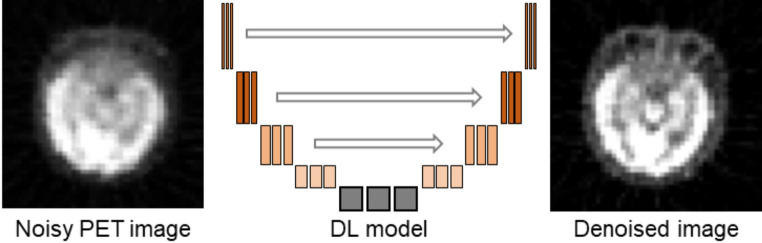
Conceptional graphic of image denoising

Nuclear medicine imaging modalities such as PET can suffer from high levels of noise and limited spatial resolution, which can hinder image clarity and reduce the accuracy of image-based dosimetry. The use of radiotracers at low doses is often attributed to these noisy low-quality images. However, increasing the injected dose raises concerns regarding increased radiation exposure in patients. Techniques such as noise reduction and super-resolution modeling can generate clinically relevant images with improved quality and detail, without compromising patient safety. Post-reconstruction image-enhancement techniques are commonly employed to improve both image quality and quantitative accuracy [58–61]. Recently, AI has emerged as a powerful tool that offers various innovative approaches for denoising (Fig. 2), deblurring, and partial-volume correction in PET imaging.

Several efforts have been made to develop DL models for enhancing nuclear medicine imaging Enhancement [62–65]. Kaplan et al. proposed a DL model to denoise low-dose PET images and estimate their full-dose equivalents by incorporating image-specific features into the loss function [66]. The model comprised two networks: an estimator and an adversarial discriminator. The estimator network included four convolutional and four deconvolutional layers with skip connections to extract and refine features from the input. The discriminator network, with one convolutional layer and one fully connected layer, classified the image patches as real or generated. The estimator was first pretrained using the mean squared error (MSE) loss, texture, and edge-preserving features, followed by the adversarial network, which further enhanced the texture and structure of the generated images. This approach results in improved image quality comparable to that of full-dose images. An unsupervised DL model was proposed by Cui et al. to perform PET image denoising using prior information from the same patient [67]. No training pairs are required because the high-quality prior image is used as the input and the noisy PET image is treated as the training label. The network learns to restore the noisy image based on the intrinsic structure of a prior image. A simulation study using the BrainWeb phantom and clinical data from PET/CT and PET/MR datasets demonstrated that the proposed method outperformed other methods in terms of contrast-to-noise ratio (CNR) improvements. Pan et al. introduced the PET consistency model (PET-CM), a diffusion-based method designed to generate high-quality full-dose PET images from low-dose inputs [68]. PET-CM uses a two-step process involving the addition of Gaussian noise in forward diffusion followed by denoising via a PET shifted-window vision transformer network in reverse diffusion. The network learns a consistency function to effectively denoise low-dose images into clean full-dose images. PET-CM outperformed state-of-the-art methods in terms of image quality and efficiency, requiring 12 times less computation time than previous models.

Traditionally, image reconstruction relies on analytical methods such as filtered backprojection (FBP) [69] or iterative algorithms such as ordered subset expectation maximization (OSEM) [70]. These methods use projection data acquired at different angles to estimate the 3D distribution of radiotracer activity. Although FBP is computationally efficient, it struggles with noise and artifacts. In contrast, OSEM incorporates corrections for attenuation, scatter, and resolution, producing higher-quality images but requiring more computational time. DL models, such as automated transform by manifold approximation (AUTOMAP), which uses a fully connected layer and CNNs [71], DeepPET using Fully Convolutional Networks (FCN) architecture [72], and a CNN iterative PET image reconstruction model using existing inter-patient information [73], have been developed. Vashistha et al. recently proposed a method that focused on reconstructing high-quality parametric PET images by integrating kinetic modeling and denoising directly into an image reconstruction pipeline [74]. This approach combines deconvolution, U-Net-based denoising, and a 1D deconvolution long short-term memory (1D-LSTM) model trained on simulated tissue time-activity curves to estimate pixel-wise kinetic parameters. Refinement was achieved using an unsupervised deep image prior. The model was tested on a brain phantom and five ^18^F-FDG PET scans; the method demonstrated high accuracy (Pearson *r* = 0.91–0.93), low error (< 0.0004), and improved CNRs compared to conventional methods.

##  Organ Segmentation

The segmentation of organs and tumors is important for dose calculations and tissue segmentation is a key parameter [75]. DL transforms organ segmentation by enabling automated pixelwise labeling with high accuracy. For example, FCNs capture spatial and contextual features, making them effective in delineating complex anatomical structures and adapting to varying shapes and sizes [52]. In this section, we discuss the advancements in DL techniques for organ segmentation.

Vavekanand et al. introduced NeuroDNet, a CNN-based model for brain tumor detection that combines 3D and 2D convolutional layers to process volumetric data from MRI scans [76]. The hybrid architecture captures both local and global tumor characteristics using multiscale convolutional layers. Trained on a large and diverse dataset of over 2,000 MR images, NeuroDNet achieved 94% validation and 92% testing accuracy. Tian et al. introduced another tumor image segmentation model, which was an improved U-Net model incorporating the GSConv module and the Efficient Channel Attention (ECA) mechanism [77]. GSConv enhances the spatial feature extraction via grouped and displaced convolutions, whereas ECA recalibrates the feature channels to focus on important details. The model was trained on 500 image-mask pairs. These improvements enabled the model to capture multiscale features and produce more accurate tumor segmentation, particularly at the tumor boundaries. The experimental results showed that the enhanced U-Net outperformed the traditional U-Net. Liao et al. introduced AbsegNet, a DL model designed to segment 16 organs [78]. Using three datasets totaling 544 CT scans from different centers, AbsegNet was trained, validated, and clinically assessed. The robustness of the model was enhanced via data augmentation and knowledge distillation. It achieved high Dice similarity coefficients (DSCs) (mean: 86.73–88.04%) and outperformed established models, such as SwinUNETR [79] and UNet [80]. Clinical evaluations showed that no revisions were required for several organs (e.g., the liver, kidneys, and spleen) and minimal revisions for others, with only 15% of patients needing major revisions for the colon and small bowel contours. Peng et al. improved the U-Net architecture by introducing a batch normalization layer, residual squeeze-and-excitation layer, and unique organ-specific loss function for DL training [81]. A total of 260 and 50 CT images were used as the training and test sets, respectively. Validation against manual delineations and STAPLE contours showed strong performance, with an average DSC of 83.75%. OrganNet achieved high accuracy in segmenting large-volume organs (84.97–95.00%) and competitive results for small-volume organs (55.46–91.56%), often surpassing manual delineation accuracy. Another study by Amjad et al. developed and evaluated five DL-based autosegmentation models for 42 organs across three major tumor sites: male pelvis (MP), head and neck (HN), and abdomen (ABD) [82]. The models, which were based on a modified 3D U-Net architecture, were trained using both general multi-institutional and custom single-institution datasets. The models used adaptive spatial resolution for small or narrow values and pseudo-scan extension for short CT scans. The custom models generally outperformed the general models, with DSCs ranging from 0.8 to 0.98 for 74%. Notably, the models showed improved accuracy for small or complex organs such as the eye lens and optic nerves. Auto-segmentation reduced the time required for manual contouring by up to 88% for MP, 80% for HN, and 65% for ABD, thereby demonstrating its clinical utility in radiation treatment planning. Several other models have been developed for medical image segmentation [83–88].

##  AI for Image-Based Dosimetry

DL has become an important tool in image-based dosimetry, increasing the speed and accuracy of the process. Voxel-based dose maps can be estimated or predicted using DL models, such as 3D CNNs, to analyze medical images and predict the distribution of radiation across voxels. In addition to direct dose estimation or prediction, the accurate segmentation of organs is crucial for dosimetry because it directly affects the precision of radiation dose calculations. DL models such as U-Net are used to automatically identify and outline organs and tumors in medical images with high precision and reduced time compared with manual work. In this section, we discuss recent DL models for dose prediction or estimation and image segmentation.

Xue et al. addressed the challenge of predicting voxel-wise absorbed-dose maps for RPT using pre-therapy PET imaging, emphasizing intra-organ heterogeneity [89]. Data from 23 patients with metastatic castration-resistant prostate cancer treated with ^177^Lu-PSMA I&T were analyzed, revealing moderate correlations between PET imaging and actual dose maps due to pharmacokinetic variability. A 3D RPT DoseGAN with a generator and discriminator was trained on 3D image patches (32 × 32 × 32) to overcome the limited training samples. The DL-based approach significantly outperformed the traditional organ dose-guided projection methods, achieving a lower voxel-wise normalized root mean squared error (0.79% vs. 1.11%, *p* < 0.05) and superior dose prediction accuracy (e.g., R² = 0.92 for the kidneys). Using SPECT/CT images, Mansouri et al. introduced a hybrid transformer-based DL model designed for voxel-level dosimetry in ^177^Lu-DOTATATE therapy to enhance the accuracy of dose map predictions [90]. The model used a multiple-VSV (MSV) approach and a modified UNet Transformer architecture, which was trained on co-registered CT images from 22 patients undergoing therapy. The model was tasked with predicting the differences between MC and MSV dose maps. Once the difference was predicted, the MC dose maps were reconstructed by adding the output to the MSV maps. The model was trained using fivefold cross-validation with 2D axial slices of CT images. The results revealed that the DL model outperformed both the MSV and SSV approaches, achieving a voxel-level relative absolute error (RAE) of 5.28 ± 1.32. The model also exhibited high gamma analysis pass rates (99.0 ± 1.2%) and significantly improved computational time, processing a single-bed SPECT scan in only 3 s (compared to 2 days for MC). Kassar et al. developed a physiologically based pharmacokinetic (PBPK)-adapted deep learning approach for the pre-therapy prediction of voxel-wise dosimetry in RPT using synthetic patient data simulating ^68^Ga-PSMA-11 and ^177^Lu-PSMA-I&T images [91]. The model was guided to produce physiologically plausible dose maps by integrating PBPK modeling into a conditional GAN. A 3D U-Net generator and custom discriminator were trained with PBPK-informed loss functions, significantly improving the dose prediction accuracy in critical organs, such as the kidneys, liver, spleen, and salivary glands, compared with purely data-driven methods. This hybrid framework demonstrates the potential of combining mechanistic models with AI to enable personalized voxel-level dose planning using single pre-therapy scans. Additional DL image-based dose estimation models are presented in Table 2. Additional DL models for dose prediction, estimation, and dose accuracy enhancement have been proposed [92–109].

**Table 2:** DL based models for dose Estimation and prediction

Author	Model/architecture	Data	Achievement
Karimipourfard et al. [110]	Pix2Pix GAN	[¹⁸F]FDG PET/CT images	The model achieved strong agreement with MC reference doses
Akhavanallaf et al. [111]	DNN (ResNET)	CT images for density maps and MC-generated voxelwise S-value	The DNN predicted S-value kernels with 4.5% MRAE compared to MC (voxel level: 2.6% and organ level: 5.1% MRAE)
Mao et al. [112]	CNN (Dw, w CNN, FiLM and 3D U-net)	CT images	Dose distributions closely matched MC simulations, (0.73% difference for prostate CTV D90 and 1.1% for rectum D2cc.)
Götz et al. [113]	DNN-EMD (hybrid U-net/EMD)	CT, Dose maps estimated according to the MIRD protocol and measured SPECT distributions of ^177^Lu radionuclei	Superior performance of the hybrid DNN-EMD method compared to MIRD DVK dose calculation
Xing et al. [114]	Hierarchically dense U-Net	CT, AAA, and AXB dose maps	Boosted AAA doses showed improved matching to the AXB doses, with an average standard deviation gamma passing rate (1 mm/1%) 97.6% (± 2.4%) compared to 87.8% (± 9.0%) of the original AAA doses
Lee et al. [115]	CNN (U-Net)	CT and PET images	High accuracy, with voxel dose rate errors of 2.54% ± 2.09%, outperforming the VSV kernel method (9.97% ± 1.79%) and OLINDA/EXM dosimetry software (34.22%)
Liu et al. [116]	Combination of a deep residual network (ResNet) and a deconvolution network (U-ResNet-D)	CT images representing the anatomical structures and their corresponding dose-related information for each slice	Reducing bias ranging from − 2.0–2.3%, prediction error between 1.5% and 4.5%

*AAA* anisotropic analytic algorithm, *AXB* Acuros XB, *CNN* Convolutional neural network, *CT* computed tomography, *CTV* Clinical target volume, *DNN-EMD* deep neural networks with empirical mode decomposition, *DVK* dose voxel kernel, *FDG* fluorodeoxyglucose, *MC* Monte Carlo, *MIRD* Medical Internal Radiation Dose, *MRAE* Mean relative absolute error, *PET* positron emission tomography

##  Discussion

This review examines the role of AI in different tasks contributing to dosimetry. Regarding image synthesis and generation, all reviewed models showed promising results. However, the statistical analysis of the image quality metric values varied for each model, potentially owing to differences in dataset size, preprocessing techniques, target region, network architectures, or loss functions. The produced image should be as similar as possible to the labeled or reference image. The images produced were evaluated based on the SSIM, PSNR, MSE, and mean absolute error (MAE), among other metrics. SSIM measures the similarity between two images by considering the luminance, contrast, and structural information, with higher values indicating better resemblance. The PSNR quantifies the ratio between the maximum possible power of a signal and the power of noise, expressed in decibels, where higher values correspond to superior image quality. The MSE calculates the average squared difference between the predicted and actual values, with lower values reflecting better image fidelity. Similarly, the MAE evaluates the average magnitude of the absolute differences between the predicted and actual values, thereby providing a clear measure of the overall prediction accuracy.

The potential of DL to generate clinically viable high-quality images should be considered. Schaefferkoetter investigated the DL transformations in medical imaging [117]. used GANs to generate synthetic CT images from whole-body MR data in PET/MR systems. This study focused on ensuring high-quality, anatomically accurate images for PET attenuation correction. The results indicated that synthetic CT images performed better than traditional MR-based methods in quantifying tracer uptake. Additionally, the synthetic CT approach showed an improved correlation with the CT-derived mu maps. Galapon et al., used MC dropout-based uncertainty maps to evaluate sCT quality for adaptive proton therapy [118], achieving high correlations (*r* = 0.92) between the uncertainty maps and Hounsfield Unit (HU) errors. Alvarez Andres et al. [119] investigated 3D CNNs for pseudo-CT (pCT) generation and revealed that larger training datasets enhanced pCT quality, whereas MRI preprocessing and sequence variations had minimal impact on dosimetric performance. Kazemifar et al. aimed to improve the dosimetric accuracy of synthetic CT images generated using a DL approach [120]. GAN was used with mutual information loss to address MRI-CT misalignment, achieving an MAE of 47.2 ± 11.0 HU and DSC of 80% ± 6% in bone structures, demonstrating its feasibility for MRI-only workflows.

The accuracy of DL for dose prediction was investigated by Götz et al. [113], who focused on kidney dosimetry in patients undergoing Lu-177-DOTATOC or PSMA therapy [121], in which the neural network predicted dose voxel kernels from density kernels derived using MC simulations. The results showed that the DL approach provided superior accuracy in predicting absorbed radiation doses compared with the traditional method, with no additional computational effort. This method has been proven highly effective in estimating radiation doses in clinical practice.

The accuracy, generalization, and robustness of DL models for organ segmentation are very important for dosimetry and should be prioritized. Koo et al. conducted a comparative evaluation of DL for autosegmentation [122]. This study involved the training of a prototype algorithm built on a combined U-Net and V-Net architecture. The model demonstrated superior accuracy across multiple evaluation metrics, including the DSC, Hausdorff distance (HD), and voxel-penalty metric. When trained with gold-standard data, the prototype achieved a DSC of 0.81, surpassing commercial models trained on the same dataset (0.74) and external data (0.66). In addition, 93% of the auto-segmented structures from the prototype model were clinically useful. However, the study also mentioned that segmentation results can vary depending on the training data and institutional differences. Therefore, the standardization and optimization of DL models for clinical use is critical.

Although DL models have shown promising results regarding dose estimation, organ segmentation, image synthesis, and image generation, several challenges remain. First, therapeutic radiopharmaceuticals are administered at low injection doses owing to their high cytotoxicity, which may cause side effects in normal tissues. Therefore, estimating the therapeutic radiopharmaceutical radiation dose based on theranostic pair data, which is a data extracted form imaging a surrogate radionuclide (surrogate imaging) that is labeled to the same targeting vehicle or with similar chemical properties as the therapeutic radionuclide, using DL can improve treatment planning by providing dose assessments while minimizing toxicity risks.

Secondly, owing to the low injection dose and patient burden, there is a high possibility of the absence of medical images. Consequently, DL models designed to estimate human dose or generating human medical images utilizing animal data and applying extrapolation methods [123] could bridge this gap by leveraging cross-species data translation.

Third, image-based dosimetry typically requires multiple post-injection scans with at least two to three time points. However, acquiring multiple scans remains challenging with the use of many radiopharmaceuticals. Therefore, DL models that generate later or earlier scans from a single timepoint scan, such as the model developed by Kim et al. [56, 57], should receive greater attention and be extended to a wider range of radiopharmaceuticals. This would contribute to more efficient imaging protocols, thereby reducing the scanning burden while maintaining dosimetric accuracy.

Fourth, some radionuclides used in RPT, such as ^213^Bi, ^177^Lu, ^211^At, and ^212^Pb, exhibit short decay chains. However, others such as ^225^Ac and ^223^Ra produce multiple daughter radionuclides that contribute to the total radiation dose and may exhibit different biodistribution patterns by breaking the bond with the parent radionuclide, owing to their recoil energy [124–126]. Therefore, a DL model that accounts for daughter radionuclide dose contributions and their in vivo behaviors is required to enhance dosimetric modeling and precise radiation dose calculations.

Fifth, in the absence of 3D images, AI models for transforming 2D into 3D images are helpful alternatives for obtaining 3D dose maps. Almeida et al. proposed a CNN-based DL model to reconstruct 3D medical image volumes from a single 2D X-ray image [127]. The model demonstrated promising performance, achieving mean SSIM scores of 0.77 ± 0.05 for knee and 0.78 ± 0.06 for hip reconstructions when compared to ground-truth CT volumes. Other models have also been proposed [128, 129], however, other modalities for 2D images, such as planar images, have been addressed using AI models.

Finally, standardization, the optimization, and rigorous evaluation of DL models is crucial for their reliable application in dosimetry. Variability in training datasets, preprocessing techniques, network architectures, and loss functions can significantly affect the performance and generalizability of DL models. Without standardization, inconsistencies across institutions or datasets may lead to suboptimal or inaccurate results that undermine the clinical utility. The optimization of DL models ensures that they are tuned to achieve the best possible outcomes in terms of accuracy, efficiency, and robustness. This is particularly important in clinical applications, such as organ segmentation or dose prediction, where precision directly affects patient safety and treatment efficacy. Establishing standardized protocols and benchmarks for training, testing, and validation fosters consistency, facilitates cross-institutional collaboration, and accelerates the clinical adoption of DL models.

##  Conclusion

The transformative role of DL in advancing personalized dosimetry for cancer treatment was reviewed. By addressing the limitations of traditional organ-level and voxel-based dosimetry methods, DL has demonstrated remarkable potential for enhancing image quality and organ segmentation, which directly contributes to the accuracy of estimated dose distributions. DL architectures, including U-Net, GANs, and transformer-based models, significantly improve the dosimetry precision and efficiency. However, key challenges persist, including the need for accurate dose prediction from theranostic pairs, addressing missing imaging data, and modeling complex radionuclide decay chains. Additionally, the standardization and optimization of DL models is essential to ensure accuracy, efficiency, and clinical reliability. Overcoming these challenges is critical to ensure their reliable and effective integration into clinical workflows.

## Data Availability

Data sharing not applicable to this review article as no data analysis.
